# Single-molecule analysis of DNA replication reveals novel features in the divergent eukaryotes *Leishmania* and *Trypanosoma brucei* versus mammalian cells

**DOI:** 10.1038/srep23142

**Published:** 2016-03-15

**Authors:** Slavica Stanojcic, Lauriane Sollelis, Nada Kuk, Lucien Crobu, Yves Balard, Etienne Schwob, Patrick Bastien, Michel Pagès, Yvon Sterkers

**Affiliations:** 1 University of Montpellier, Faculty of Medicine, Laboratory of Parasitology-Mycology, Montpellier, F34090, France; 2 CNRS 5290 - IRD 224 - University of Montpellier (UMR “MiVEGEC”), Montpellier, F34090, France; 3 University Hospital Centre (CHU), Department of Parasitology-Mycology, Montpellier, F34090, France; 4 Institute of Molecular Genetics, CNRS UMR5535 & University of Montpellier, Montpellier, F34293, France

## Abstract

*Leishmania* and *Trypanosoma* are unicellular parasites that possess markedly original biological features as compared to other eukaryotes. The *Leishmania* genome displays a constitutive ‘mosaic aneuploidy’, whereas in *Trypanosoma brucei*, the megabase-sized chromosomes are diploid. We accurately analysed DNA replication parameters in three *Leishmania* species and *Trypanosoma brucei* as well as mouse embryonic fibroblasts (MEF). Active replication origins were visualized at the single molecule level using DNA molecular combing. More than one active origin was found on most DNA fibres, showing that the chromosomes are replicated from multiple origins. Inter-origin distances (IODs) were measured and found very large in trypanosomatids: the mean IOD was 160 kb in *T. brucei* and 226 kb in *L. mexicana*. Moreover, the progression of replication forks was faster than in any other eukaryote analyzed so far (mean velocity 1.9 kb/min in *T. brucei* and 2.4–2.6 kb/min in *Leishmania*). The estimated total number of active DNA replication origins in trypanosomatids is ~170. Finally, 14.4% of unidirectional replication forks were observed in *T. brucei*, in contrast to 1.5–1.7% in *Leishmania* and 4% in MEF cells. The biological significance of these original features is discussed.

In all eukaryotes, DNA replication is a tightly regulated process that initiates from multiple replication origins that are distributed along the chromosomes. Accurate regulation of DNA replication ensures faithful transmission of eukaryotic genomes and maintenance of genome ploidy and chromatin organization^1^. DNA replication is under the control of genetic and epigenetic mechanisms and is closely linked to the functional organization of the nucleus^2^. In most eukaryotes, origins are not conserved sequences but appear to be defined by DNA structure and chromatin environment^2^. An excess of potential replication origins is made before the S phase of the cell cycle, through the assembly of pre-replication complexes. However, only a subset of these potential origins is activated during S phase to initiate DNA synthesis. Flexibility in the choice of replication origins is a general feature of both yeast and metazoan replication origins, but the choice is surprisingly plastic and opportunistic^3^.

Conventional eukaryotic models are actually closely related in the evolutionary time scale^4^ and more ‘divergent’ eukaryotes might well have different DNA replication mechanisms. Trypanosomatids branched very early in eukaryotic history^4^ and they have attracted attention as original models for biological and comparative studies. *Trypanosoma brucei* and *Leishmania* sp. are protozoan parasites responsible for some of the most debilitating diseases in humans and livestock such as leishmaniasis, sleeping sickness and nagana. Despite the strong synteny observed between the genomes of these two trypanosomatids, their organization is very different. The genome of *T. brucei* is composed of 11 megabase-sized chromosomes ranging from 1 to 6 Mbp in length, 3–5 intermediate chromosomes of 200–500 kb and ~100 mini-chromosomes ranging from 30 to 150 kb^5^,^6^. In contrast, the genome of *Leishmania* comprises 36 chromosomes for the Old World species, hereafter *L. major* and *L. donovani*, and 34 for the New World species, hereafter *L. mexicana*^7^,^8^, ranging from 0.3 to 3 Mb in size. In all trypanosomatids, genes are organized in large unidirectional polycistronic units (so-called “directional gene clusters” or DGCs), made of many functionally non-related genes present on the same DNA strand. Neighbouring transcription units can be either convergent or divergent, and the regions between them are known as strand switch regions (SSRs). Divergent SSRs have been associated with the initiation and convergent ones with the termination of transcription^9^,^10^. Both types of SSRs have also been shown to contain origins of replication^11^,^12^ as well as centromeric regions^13^ in *T. brucei* but not *Leishmania*. Another unique feature is the constitutive ‘mosaic aneuploidy’ demonstrated by Fluorescent *in situ* hybridization (FISH) data in *Leishmania* strains^14^,^15^, contrasting with the diploidy of *T. brucei* established for its 11 ‘megabase’ chromosomes^16^. The biological significance of mosaic aneuploidy in *Leishmania sp.* remains ill defined, but some insights were obtained by studying the distribution of chromosomes during mitosis. FISH monitoring of the sister chromosomes segregation during mitotic division showed that *Leishmania* display high rates of asymmetric chromosome allotments, and that all dividing nuclei totalized an odd number of chromosome homologues^14^,^17^. An even number of chromosome homologues in dividing cells indicates chromosome segregation defects^18^,^19^, while an odd number rather suggests an unconventional regulation of DNA replication; this would allow over- and under-replication events leading to either the complete re-duplication or the absence of a given chromosome at the end of the cell cycle. The regulation of DNA replication is thought to be essential for the occurrence and persistence of mosaic aneuploidy in *Leishmania*^14^,^17^ and might also be responsible for a laboratory-induced, well tolerated aneuploidy in *T. brucei*^20^. Here, we analysed DNA replication in three *Leishmania* species (promastigote forms of *L. donovani, L. major* and *L. mexicana*) and in the related *T. brucei* (procyclic forms) at the single molecule level, using state-of-the-art DNA molecular combing. This technique allows uniform stretching of DNA fibres, hence accurate measurements of inter-origin distances (IODs) as well as of the symmetry and velocity of progressing replication forks^21^. We show here that DNA replication in these parasites exhibit highly original features. The inter-origin distances are very large and DNA replication progresses more rapidly, particularly in *Leishmania*, than in any other eukaryotic cells analysed so far. The estimated number of active origins was similar in all trypanosomatids (150–180). The major difference in DNA replication dynamics between these parasites is the high occurrence of unidirectional replication forks in *T. brucei*. To our knowledge, this is the first study of replication dynamics in *Leishmania*, the first one to use DNA molecular combing in trypanosomatids, and the first one to compare the replication dynamics of so-called ‘divergent’ eukaryotes and higher eukaryotes in the same experiments.

## Results

### Setting up the conditions for DNA replication analysis in trypanosomatids by DNA molecular combing

**Figure 1:**
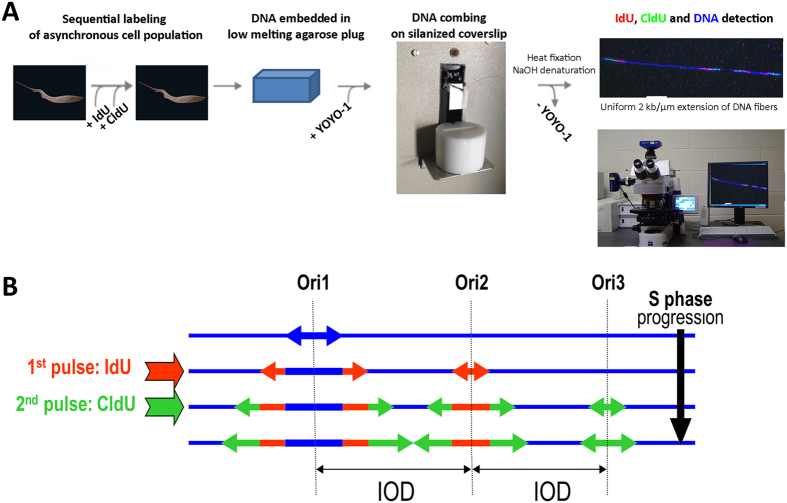
Schematic representation of the experimental approach and different signal patterns observed after two-pulse labelling of replicating DNA. (**A**) Asynchronous cells were successively labelled with equal pulses of iodo-deoxyuridine (IdU) and chloro-deoxyuridine (CldU) and chromosomal DNA combed on silanized coverslips. (**B**) Three major types of signal patterns may be observed after molecular combing and immuno-detection of the modified nucleosides, depending whether DNA replication initiates before the first pulse (Ori 1), during the first pulse (Ori 2) or during the second pulse (Ori 3). DNA fibres are counterstained in blue; IdU tracks, corresponding to the incorporation of the modified nucleotide during the first pulse, are revealed in red, whereas CldU tracks corresponding to the second pulse are in green. Arrows represent the direction of the replication forks progression, which is visually detected as a transition from red to green labeling. DNA replication initiation sites (active origins or Ori) stand in the centre of the bidirectional replication fork. The inter-origin distance (IOD) was determined by measuring centre to centre distance between two adjacent progressing bidirectional forks.

Exponential cultures of asynchronously growing cell populations of each trypanosomatid were labelled with two consecutive pulses of IdU and CldU, DNA fibres were then purified and combed onto silanized coverslips (Fig. 1A; see Material and Methods). This procedure generated long, parallel DNA fibres, with a uniform extension of 2 kb/μm^22^. The incorporated modified nucleosides were immunodetected and DNA was also systematically counterstained to ensure that (i) replication signals belonged to the same fibre, (ii) two fibres were not overlapping and (iii) the signals were not discontinuous (see Material and Methods). After incorporation of two modified nucleotides into newly replicated DNA, at least three different signal types can be expected (Fig. 1B). Several concentrations of modified nucleosides, iodo-deoxyuridine (IdU) and chloro-deoxyuridine (CldU), were tested for labelling trypanosomatids, and a minimal concentration of 300 μM was found necessary to obtain satisfactory signals of newly replicated DNA fibres. Since these concentrations were 10× higher than those usually used to label cells for DNA combing analysis, we used mouse embryonic fibroblasts (MEF cells), labelled under exactly the same conditions as a control. The analysis of DNA replication in MEF cells by molecular combing has been detailed elsewhere^23^,^24^. This allowed us to check that speed and other parameters of DNA replication were not altered by the high concentrations of modified nucleosides.

### Inter-origin distances in trypanosomatids

**Figure 2:**
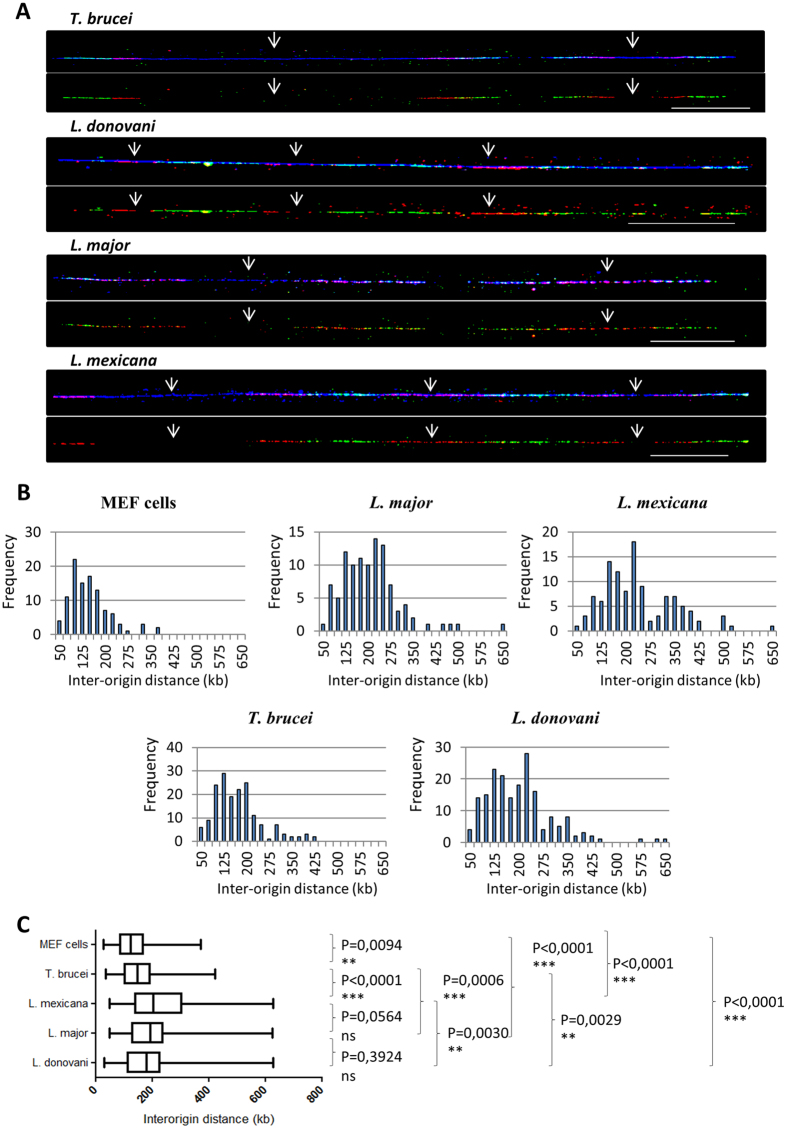
Single-molecule analysis of the inter-origin distances in trypanosomatids using molecular combing. (**A**) Representative chromosome fibres from trypanosomatids used for the analysis of inter-origin distances (IOD). Positions of the presumed origins are indicated with arrows. Scale bar: 50 kb. For each species, upper panels: counterstaining of DNA fibres is in blue, first pulse nucleosides (IdU) in red and second pulse nucleosides (CldU) in green; lower panels show the tracks from the first and second pulse nucleosides extracted from the upper panel. (**B**) Statistical distribution (frequency) of IODs in MEF cells and four trypanosomatids. (**C**) Comparative analysis of IODs in MEF cells and four trypanosomatids obtained after one-way ANOVA analysis of variance. Boxes present 25–75% range. Whiskers present minimum–maximum range. The two-tailed Mann-Whitney test was used to compare the median values of IOD between each two samples and to calculate the corresponding P values (P < 0.05 was taken as significant).

Inter-origin distance (IOD) is defined as the length between two adjacent replication initiation sites and can be determined by measuring centre-to-centre distances between two adjacent progressing forks (Fig. 1B). Representative chromosome fibres from this analysis are shown in Fig. 2A, with the position of the presumed DNA replication origins. The distributions of all IODs measured in MEF cells and four trypanosomatids are presented in Fig. 2B. More than 100 IODs were measured for each of the five cell lines studied (Table 1). The mean IOD was calculated as 136 kb (median 125 kb, see Table 1) in MEF cells, which is in agreement with the previously published data^23^,^24^. The mean IOD in *T. brucei* was 160 kb (median 149 kb). The mean IODs in *L. donovani*, *L. major* and *L. mexicana* were 189 kb (median 180 kb), 195 kb (median 193 kb) and 226 kb (median 203 kb), respectively (Table 1). The range of IODs was particularly large in *Leishmania* species where they fluctuated between 30–628 kb, 48–625 kb, 49–628 kb in *L. donovani, L. major and L. mexicana,* respectively (Fig. 2B, Table 1). In *T. brucei* the range of IOD variations was smaller than in *Leishmania* (37–422 kb) but greater than in MEF cells (27–372 kb). Sample size, fibre length and DNA counterstaining are crucial parameters to gain robust information about replication dynamics; in particular IOD measurements can be biased by DNA fibre length^24^. We therefore verified the length classes of combed DNA fibres for MEF cells, *T. brucei* and the three *Leishmania* species. The distribution of DNA fibre lengths was very similar in all five samples (Supplemental Fig. 1), showing that IOD comparisons among trypanosomatids and MEF cells is not biased by DNA fibres length. Statistical analysis of these measurements confirmed that IODs are significantly larger in *Leishmania* than in other cell types, but also larger in *T. brucei* than in MEF cells (Fig. 2C).

### Velocity of replication forks in trypanosomatids

**Figure 3:**
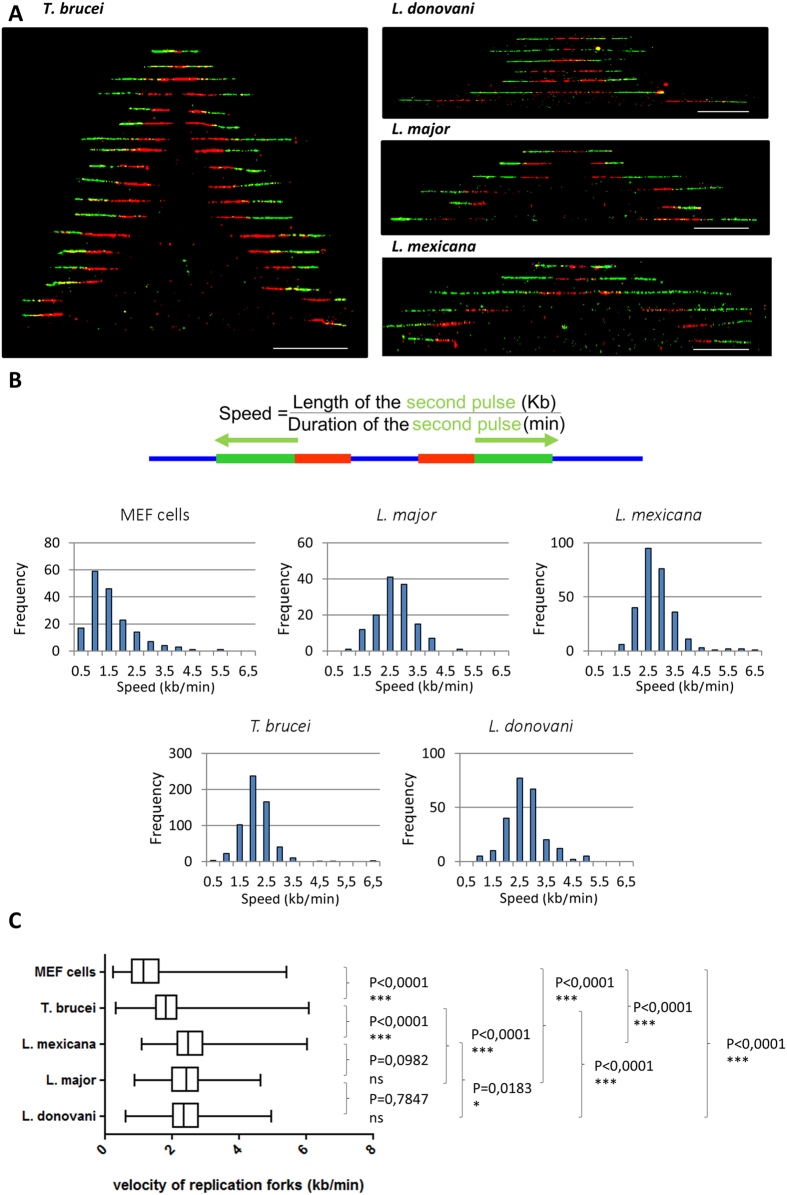
Single-molecule analysis of the velocity of replication forks in MEF cells and four trypanosomatids using DNA molecular combing. (**A**) Representative bidirectional replication forks taken from different microscopic fields were artificially assembled and centred on the position of the presumed origins. Red tracks: IdU, green tracks: CldU. Counterstaining of DNA was performed but is not shown here for the sake of clarity. Scale bar: 50 kb. (**B**) Statistical distribution of replication forks velocity measurements in MEF cells and four trypanosomatids. The velocity of the replication fork was calculated by dividing the length of the ‘green’ tracks with the duration of the second pulse. (**C**) Comparative analysis of the velocity of replication forks in the five cell lines obtained after one-way ANOVA analysis of variances. Boxes present 25–75% range. Whiskers present minimum–maximum range. The two-tailed Mann-Whitney test was used to compare the median values of the velocity of replication forks between each two samples and to calculate the corresponding P values (P < 0.05 was taken as significant).

To calculate and compare the velocities of replication forks in these five eukaryotic cell types, we divided the length of the second (CldU) labelling track by the duration of the second nucleoside pulse (see Fig. 1B). Representative chromosome fibres used for this analysis are shown in Fig. 3A, where only signals from progressing bidirectional forks are presented (IdU and CldU labeling). The distributions of >100 fork velocity measurements for all five cell types are presented in Fig. 3B and Table 1. In MEF cells, labelled under exactly the same conditions as trypanosomatid cells, the detected mean velocity was 1.36 kb/min (median 1.06 kb/min), which is in agreement with previous reports using lower concentrations of modified nucleosides^23^,^24^. In *T. brucei*, the mean replication fork velocity was 1.87 kb/min (median 1.83 kb/min), with extremes ranging from 0.34–6.09 kb/min. In *Leishmania*, these figures were 2.43 (range 0.88–4.65) kb/min, 2.45 (range 0.62–4.97) kb/min and 2.59 (range 1.11–6.03) kb/min in *L. major*, *L. donovani* and *L. mexicana* respectively. Comparative statistical analysis of fork velocities in the four trypanosomatids and MEF cells confirmed that DNA replication progressed at a similar velocity in the three *Leishmania* strains, and significantly faster in *Leishmania* than in *T. brucei* and MEF cells (Fig. 3C).

**Figure 4:**
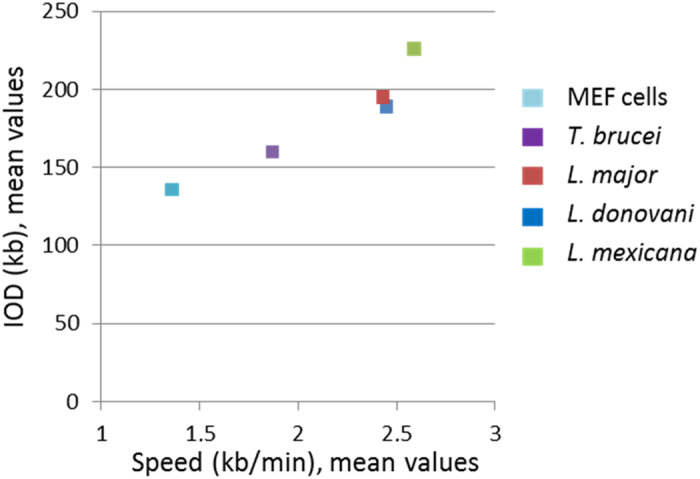
Positive correlation between mean inter-origin distances (IODs) and fork velocities. 2-way representation of mean values of fork velocities and IODs in MEF cells, *T. brucei* and three *Leishmania* species.

The interdependence of IODs and fork velocities was also analysed in these five eukaryotic cell types (Fig. 4). The mean fork velocities and IOD values tend to be positively correlated (coefficient of determination R^2^ = 0.903): the longer IODs observed in *Leishmania* cells result in faster fork progression. Similarly, the IODs were significantly shorter in *T. brucei* than in *Leishmania*, and the progression of replication forks was slower. The IODs were the shortest, and the progression of replication forks the slowest, in MEF cells.

### DNA replication dynamics in trypanosomatids

**Figure 5:**
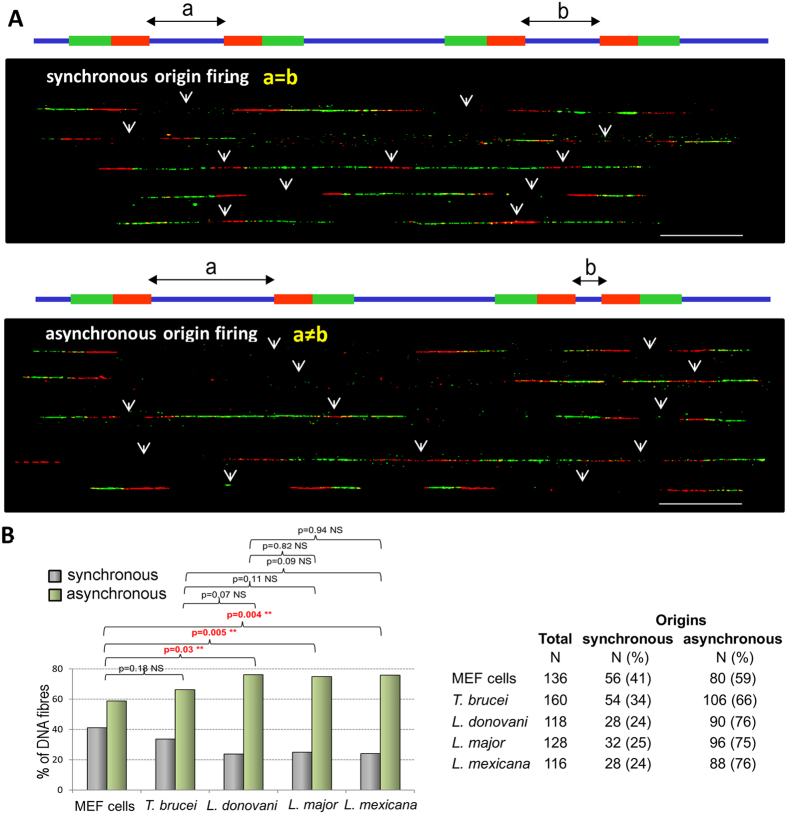
Analysis of synchronous and asynchronous adjacent origin firing. (**A**) Representative chromosome fibres used for the analysis of DNA replication dynamics showing synchronous (upper picture) and asynchronous (lower picture) firing of adjacent origins (arrows). Diagrams: “a” and “b” represent the lengths of the tracks of non-labelled DNA in two adjacent origins. They are used to discriminate between synchronous and asynchronous origins: if “a = b” the two adjacent origins are synchronous, if “a ≠ b”, they are asynchronous. (**B**) The number of DNA fibres bearing synchronous and asynchronous adjacent origin firing was counted in the five cell types (MEF cells, *T. brucei* and three *Leishmania* species). Numbers and percentages are presented in the table (right) and the percentages of each type of firing represented as histograms (left). The Chi-square test was used to calculate the P values (P < 0.05 was taken as significant).

**Figure 6:**
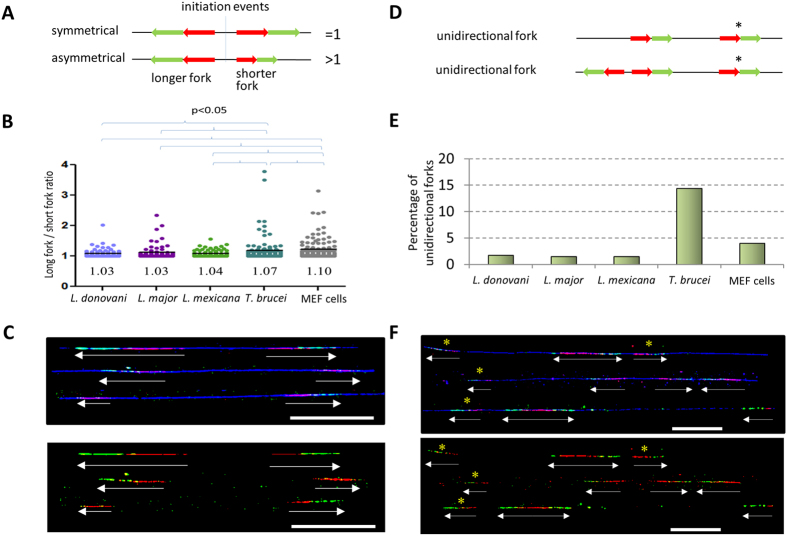
Analysis of the symmetry of progressing bidirectional replication forks. (**A**) Presentation of the concept of long fork/short fork ratios. The long fork/short fork ratio corresponds to the ratio of the longer IdU + CldU signal over the shorter IdU + CldU signal of bidirectional replication forks. A ratio >1 indicates fork asymmetry, while a ratio =1 indicates symmetry (see Materials & Methods). (**B**) Distribution of long fork/short fork ratios in MEF cells, T*. brucei* and three *Leishmania* species. Median (black bar) values are indicated below the distribution clouds. Statistically significant differences are indicated; p values were calculated using the two-tailed Mann-Whitney test. (**C**) Representative pairs of asymmetric replication forks from *T. brucei*. The forks were assembled from different microscope fields. Arrows indicates the lengths of the bidirectional forks analysed. Red tracks: IdU, green tracks: CldU, blue tracks: DNA. DNA counterstaining is not shown in the bottom panel. Scale bar: 50 kb. (**D**) Schematic presentation of the unidirectional replication forks that were analysed. Forks indicated by asterisks are counted as unidirectional forks and used in the calculation of percentage of unidirectional forks. In the top fibre, the left fork was not included for calculation as one cannot distinguish between a unidirectional fork and a fibre broken between the bidirectional forks. (**E**) Histograms showing the percentage of unidirectional replication forks over the total number of analysed forks in MEF cells, *T. brucei* and three *Leishmania* species. (**F**) Representative DNA fibres from *T. brucei* showing unidirectional forks. The fibres were assembled from different fields. Arrows indicate the direction of replication forks. Unidirectional forks indicated by asterisks were used for this analysis. Same color code and legend as in (**C**).

To further describe the replication dynamics, we analysed (i) the temporal order of adjacent origin firing, (ii) the symmetry of bidirectional replication forks’ progression and (iii) the presence of unidirectional replication forks. Temporal regulation of origin activation allows adjacent DNA replication origins firing either synchronously, when two or more neighbouring origins fire at the same time, or asynchronously when adjacent origins fire at different time points. One potential role for this regulation is to minimize fluctuations in S phase duration and to avoid persistence of unreplicated DNA in mitosis^25^. We found both synchronous and asynchronous firing of adjacent DNA replication origins (Fig. 5A). The replication origins can be activated in an asynchronous manner (Fig. 5A, lower panel) when one replication origin is activated in between two previously activated origins. Asynchronous firing of adjacent origin was observed more frequently in trypanosomatids than in MEF cells (Fig. 5B) and among the trypanosomatids, more frequently in *Leishmania* species than in *T. brucei*. In *Leishmania* species, asynchronous adjacent origin firing was three times more frequent than synchronous adjacent origin firing. The symmetry of progressing replication forks, a parameter used to estimate the occurrence of fork stalling, was also studied. Here again, MEF cells were used as a control since the analysis of replication fork symmetry in these cells was published previously^24^. The forks symmetry can be estimated as a ratio of the lengths of the longer over the shorter progressing forks that are emanating from a given origin. Progressing forks are symmetric when this ratio is equal to 1, and asymmetric when it is >1 (Fig. 6A and Methods). The distribution of these ratios in all cell types studied is shown in Fig. 6B. Asymmetric replication forks were present in MEF cells as reported previously^24^, with a median ratio of 1.10 (Fig. 6B and Table 1). Fork asymmetry values were uniform in *Leishmania* species, with median values of 1.02, 1.03 and 1.04 for *L. donovani*, *L. major* and *L. mexicana*, respectively. On the other hand, *T. brucei* was significantly more enriched in asymmetric forks (median 1.07) than *Leishmania* (Fig. 6B). Some examples of progressing replication bidirectional forks with high asymmetry, observed in *T. brucei*, are shown in Fig. 6C. The presence of asymmetric or unidirectional replication forks has to be analysed separately because the latter were excluded from the calculation of the progressing forks ratio. A schematic presentation of the analysed unidirectional replication forks is shown in Fig. 6D. The total number of unidirectional replication forks was counted, avoiding the artefacts of broken DNA fibres (Fig. 6D). The percentage of unidirectional forks over the total number of replication forks analysed was calculated in all five cell types (Fig. 6E). This was highest (14.4%) in *T. brucei*, as opposed to MEF cells, where only 4% of unidirectional replication forks were observed, and *Leishmania*, *where* unidirectional replication forks were rarely seen (1.7%, 1.5%, and 1.5% for *L. donovani*, *L. major* and *L. mexicana*, respectively). Some examples of unidirectional replication forks in *T. brucei* are presented in Fig. 6F.

## Discussion

In the present study, we examined the DNA replication dynamics in three *Leishmania* species and *T. brucei* in comparison with mouse embryonic fibroblasts (MEF). For this purpose, we used DNA molecular combing on asynchronously cultivated cells which allowed us to follow the DNA replication dynamics in cells at different time points of the S phase. Molecular combing allows monitoring the replication origins activation and the progression of replication forks, hence studying the real-time dynamics of genome replication, at a resolution of 2–5 kb and with the possibility to study >1 Mb-long DNA fibres^22^. It investigates the dynamics of DNA replication at the level of individual molecules, as opposed to high*-*throughput genomic approaches, which provide compiled replication patterns of the whole cell population.

This study showed that DNA replication forks are moving fast in trypanosomatids, especially in *Leishmania* species, compared to other eukaryotes (Fig. 3B and Table 1). Indeed, most reported mean velocities of replication forks in eukaryotes vary from 0.8 kb/min in *Drosophila* Kc cells^23^ to 1.4–1.5 kb/min in mouse embryonic stem cells, human primary normal keratinocytes, and Chinese hamster embryonic fibroblasts (CHEF); and the highest replication fork speed ever reported was 1.9 kb/min in transformed JEFF lymphoblastoid cells^24^. We found a DNA replication velocity of 2.4–2.6 kb/min in the three *Leishmania* species and 1.9 kb/min in *T. brucei*. Several factors play important roles in fork progression rates, among which the intrinsic properties of the DNA polymerase^26^, the chromatin structure and condensation state^27^, and the concentration of the dNTPs^28^,^29^. In trypanosomatids, all canonical histones homologues can be found in the genome^30^,^31^,^32^, but the patterns and types of histone modifications show substantial differences when compared to higher eukaryotes^33^,^34^,^35^,^36^. Chromatin is classically packaged around histones, but the chromosome condensation state is more relaxed in these organisms than in higher eukaryotes. A recent study of replication velocity in *T. brucei* using another technique named ‘single molecule analysis of replicated DNA or SMARD’, yielding spread DNA rather than combed fibres, found even higher replication velocities in procyclic and bloodstream forms (mean 3.7 ± 0.1 and 4.4 ± 0.1 kb/min, respectively)^38^. We consider our data more accurate, because (i) DNA molecular combing enables the linearization of long DNA fibres and their uniform extension at 2 kb/μm; (ii) the DNA fragments obtained using SMARD are much smaller, which did not allow measuring IODs, (iii) no control cells were used in the SMARD study. In addition, the authors did not specify that they put the cells on ice after labelling, and we experimentally determined that, if this is not done, DNA replication continues, yielding artificially high replication fork velocities, e.g. 3.8 kb/min in *L. donovani* (data not shown).

Molecular combing also allows measuring the distances between active origins, or IODs, which have never been reported for divergent eukaryotes. IODs in *T. brucei* (mean 160 kb) and *Leishmania* (mean 226 kb for *L. mexicana*) were significantly larger than those reported in the literature for mammalian cells, *e.g.* 111 kb, 136 kb, 139 kb and 147 kb, in human primary keratinocytes, MEF cells, mouse embryonic stem cells and JEFF cells, respectively^23^,^24^,^39^. IODs in trypanosomatids were also much larger than those observed in insect cells (*Drosophila* Kc cells)^23^, *Arabidopsis thaliana*^40^ and budding yeast^41^,^42^ where the average IODs are 73 kb, 77 kb and 46 kb, respectively. One explanation for this uncommon feature might be the highly specific gene organization in trypanosomatids as large directional gene clusters. In addition, we observed a positive correlation between fork velocity and IODs in the four trypanosomatids and MEF cells (Fig. 4D): the larger was the mean IOD in one cell type, the faster was the progression of replication forks. A similar correlation was previously described for lymphoblastoid cells and fibroblasts^43^ and it can be also inferred from previously published data in other cell types^22^,^23^,^24^. This tendency suggests that fork velocity and IOD are co-regulated by a mechanism that ensures the replication of the entire genome during the cell cycle. One may consider that this mechanism is universal and conserved throughout evolution.

It is possible to infer the number of active origins per haploid genome from our data (Table 1). Dividing the size of the haploid genome of *T. brucei*, *i.e.* 26 Mb^30^, by the mean IOD of 160 kb (or the median 149 kb), we inferred the presence of ~170 (163–175) active origins per haploid genome in this organism. The same calculation applied to *L. major* and *L. donovani* (genome size 32.8 Mb and 32.4 Mb, respectively^44^,^45^) yields an estimate of 168–180 active origins per haploid genome; and a slightly smaller number of active replication origins was computed for *L. mexicana* (around 150). These figures have to be compared with the 42 early firing DNA replication origins that were recently mapped in the *T. brucei* genome using genome-wide marker frequency analysis coupled with deep sequencing (MFAseq), which compares DNA read depth in replicating cells *versus* non-replicating cells^46^, and with the ~100 active origins predicted by these authors by extrapolation^11^. The discrepancy between these two estimates of active origin numbers still appears paradoxical because genome-wide analysis should overestimate the number of active origins as it compiles all origins from the cell pool (reviewed in^47^). The SMARD analysis^38^ supported the presence of more, unmapped, replication origins in *T. brucei* but without estimation of a final origin number. Therefore, our results bring a more precise insight on the number of active origins in trypanosomatids.

One of the major findings of our study is the presence of multiple active origins on the chromosomes of *Leishmania*. Indeed, MFAseq has also recently been applied to two *Leishmania* species (*L. major* and *L. mexicana*)^48^ and found that each chromosome of *Leishmania* appears to be replicated from a single origin. Our data contradict this statement. We were able to see more than one active origin on most DNA fibres in all three analyzed *Leishmania* species, like in *T. brucei* (Figs 2A and 5A) and any other eukaryote analyzed so far. Incidentally, in their discussion, the authors raised the issue that their data may not be relevant for *Leishmania* biology and that a single-origin chromosome is not consistent with the duration of the S phase estimated at 2.9 h in *L. mexicana*^49^. Indeed, considering the mean fork velocity of 2.6 kb/min found here for *L.mexicana* (Table 1), the replication of the largest chromosomes (1–3 Mb) from a single origin would take 6–20 hours. The robustness of our approach clearly shows that the *Leishmania* chromosomes, at least the largest ones, are replicated from multiple origins.

The most distinguishing feature of DNA replication in *T. brucei as* compared to *Leishmania* and MEF cells is the high frequency of unidirectional replication forks. The reason for such a discrepancy between the two parasites is unknown. However, it is tempting to implicate the numerous ‘mini-’ and ‘intermediate’ chromosomes of *T. brucei*, since these are specific of *T. brucei* and built around repetitive palindromes^50^; the latter have been proposed to adopt hairpin structures during lagging-strand synthesis, which represent obstacles to fork progression^51^. Similarly, collisions between transcription and replication can also cause replication fork stalling. Using MFA seq, Tiengwe *et al.*^11^ observed asymmetric replication peaks within directional gene clusters (DGCs) on megabase-sized chromosomes and noted that replication was slower when transcription was progressing in the direction opposite to DNA replication. However the organization of the genome in DGCs is not a specificity of *T. brucei* but a feature shared by all trypanosomatids. Finally, they did not observe unidirectional replication peaks.

In conclusion, DNA molecular combing here revealed and clarified original features of DNA replication in trypanosomatids in comparison with other eukaryotic model organisms, such as large IODs, fast replication fork progression and, in *T. brucei*, the elevated occurrence of unidirectional replication forks. The characteristics of DNA replication in these either aneuploid or diploid organisms are often similar but also show differences. Yet, these are not sufficient to explain their differences in ploidy patterns. Therefore, rather than to different replication parameters, it is probable that the mechanisms responsible for the unique mosaic aneuploidy of *Leishmania* are related to a ‘leaky’ regulation of DNA replication, still to be characterized, and most likely at the level of the licensing or re-licensing of DNA replication origins.

## Methods

### Parasites and *in vitro* culture

Promastigotes of *Leishmania major* ‘Friedlin’ (MHOM/IL/81/Friedlin), *L. mexicana* (MHOM/GT/2001/U1103, clone 25), and *Leishmania donovani* (LD1S) were grown at 26 °C in RPMI1640 (Gibco BRL) supplemented with 10% fetal bovine serum (FBS)^52^. Procyclic forms of the Lister 427 wild type strain and Lister 427 29–13 cell line of *T. brucei* were grown at 27  °C in SDM-79 (PAA Laboratories) supplemented with 10% FBS and 7 μg/ml hemin, and with 30 μg/ml of hygromycin and 10 μg/ml of geneticin for the 29–13 line^53^.

### DNA molecular combing

Asynchronous insect form cell populations of *T. brucei* and three *Leishmania* species (*L. donovani* Ld1S, *L. major* Friedlin 3171 and *L. mexicana* U1103) were grown to the density of 4.10^6^ cells/mL and sequentially labelled with two modified nucleosides, iodo-deoxyuridine (IdU, Sigma) and chloro-deoxyuridine (CldU, Sigma). Cells were sequentially labelled with 300 μM IdU and 300 μM CldU for 20 (or 15) minutes each, without intermediate wash. After labeling, the cells were immediately placed on ice to stop DNA replication. MEF cells derived from 13.5-d mouse embryos were cultured as previously described^54^ and used at passage 4–5. They were labelled with IdU and CldU in the same manner and with the same concentrations of modified nucleosides as parasite cells. Cells were then centrifuged (3000 g for 5 min at +4 °C) and washed once with 1× phosphate-buffered saline (PBS); 1.10^8^ cells were resuspended in 100 μl of 1× PBS with 1% low-melting agarose in order to embed cells in agarose plugs. Plugs were incubated in 0.5 mL of 0.5 M EDTA with 1% N-lauryl-sarcosyl and 2 mg/mL proteinase K and incubated at 45 °C for 2 days (fresh solution was added on the second day). Complete removal of digested proteins and other degradation products was performed by washing the plugs in 0.5 M EDTA and TE buffer several times. Protein-free DNA plugs were then stored in 0.5 M EDTA at 4 °C or used immediately for combing. Agarose plugs were stained with YOYO-1 fluorescent dye (Molecular Probes) in TE buffer for 1 h, washed with TE buffer, resuspended in 100 μl of TE buffer and melted at 65 °C for 15 minutes. The solution was maintained at 42 °C for 15 minutes and treated overnight with β agarase (Sigma Aldrich). After digestion, 4 ml of 50 mM MES (2-(N-morpholino) ethanesulfonic acid, pH 5.7) were added very gently to the DNA solution and then DNA fibres were combed and regularly stretched (2 kb/μm) on silanized coverslips as described previously^22^. Combed DNA was fixed at 65 °C for 2 hours, denatured in 1N NaOH for 20 minutes and washed several times in PBS. After denaturing, silanized coverslips bearing the DNA fibres were blocked with 1% BSA and 0.1% Triton X100 in PBS. Immuno-detection was done with antibodies diluted in 1 × PBS, 0.1% Triton X100, 1% BSA and incubated at 37 °C in a humid chamber for 60 min. Each step of incubation with antibodies was followed by extensive washes with 1 × PBS. Immuno-detection was done with anti-ssDNA antibody (1/100 dilution, Chemicon), the mouse anti-BrdU antibody (1/20 dilution, Becton Dickinson) and a rat anti-BrdU antibody (1/20 dilution, Sera Lab) that recognize the IdU and CldU tracks, respectively. The mouse anti-BrdU, clone B44, is derived from hybridization of mouse Sp2/0-Ag14 myeloma cells with spleen cells from BALB/c mice immunized with iodouridine-conjugated ovalbumin. It reacts with iodouridine and BrdU^55^,^56^. The rat anti-BrdU antibody, clone BU1/75 (ICR1), cross reacts with chlorodeoxyuridine (CldU) but does not cross react with thymidine or iododeoxyuridine^56^. The secondary antibodies were goat anti-rat antibody coupled to Alexa 488 (1/50 dilution, Molecular Probes), goat anti-mouse IgG1 coupled to Alexa 546 (1/50 dilution, Molecular Probes), and goat anti-mouse IgG2a coupled to Alexa 647 (1/100 dilution, Molecular Probes). Coverslips were mounted with 20 μl of Prolong Gold Antifade (Molecular Probes), dried at room temperature for 2 hrs and processed for image acquisition using a fully motorized Leica DM6000 microscope equipped with a CoolSNAP HQ2 1 CCD camera and controlled by MetaMorph (Roper Scientific). Images were acquired with a 40× objective, where 1 pixel corresponds to 322 bp. Thus, at a magnification of 40×, one microscope field corresponds to ~450 kb. Observation of longer DNA fibres therefore requires the capture of adjacent fields or the use of a fluorescence microscope equipped with a motorized stage that enables scanning of slides with high precision. Fibres <150 kb were excluded from the analysis. Inter-origin distances and the velocity of replication forks were measured manually using the MetaMorph software. Statistical analyses of inter-origin distances and velocities of replication forks were performed using Prism 5.0 (GraphPad). At least three independent cell labeling and combing experiments were performed for each analysis presented here.

### Statistical analysis

Replication fork speed was estimated on individual forks displaying an IdU track flanked by a CldU track. Only intact forks were recorded as ascertain by DNA counterstaining. The estimations of fork asymmetry were calculated as the ratio of the longer track over the shorter track in progressing bidirectional forks. A longer fork/shorter fork ratio >1 indicates asymmetry. Inter-origin distances were measured as the distance (kb) between the centers of two adjacent progressing forks located on the same DNA fibre. The GraphPad Prism (GraphPad Software) was used to generate graphs and perform statistical analysis. DNA replication parameters generally do not display a Gaussian distribution^57^. Statistical comparisons of the distributions were therefore assessed using the nonparametric Mann–Whitney rank sum test. Two-tailed tests were systematically used. The number of synchronous and asynchronous origins in the different cell types were compared using Chi-square tests. Statistical significance was set at P ≤ 0.05. The correlation between fork speed and IODs was analysed using linear regression and the coefficient of determination R^2^.

## Additional Information

**How to cite this article**: Stanojcic, S. *et al.* Single-molecule analysis of DNA replication reveals novel features in the divergent eukaryotes *Leishmania* and *Trypanosoma brucei* versus mammalian cells. *Sci. Rep.* **6**, 23142; doi: 10.1038/srep23142 (2016).

## Supplementary Material

Supplementary Information

## Figures and Tables

**Table 1:** DNA replication parameters in three *Leishmania* species, *T. brucei* and MEF cells.

	*L. donovani*	*L. major*	*L. mexicana*	*T. brucei*	MEF cells
**Inter-origin distance (kb)**					
Median (kb)	179.7	192.7	203.3	148.8	124.6
Mean (kb)	188.5	194.8	226	159.9	135.8
Minimum (kb)	29.5	48.6	49.3	36.7	27.4
Maximum (kb)	628.1	625.4	628.1	421.6	372.3
25% Percentile (kb)	114.2	128.2	140.7	103.8	86.37
75% Percentile (kb)	228.3	237.5	306.4	192.4	168.1
Number of values measured	189	130	118	172	140
**Velocity of replication forks (kb/min)**					
Median (kb/min)	2.37	2.45	2.48	1.84	1.16
Mean (kb/min)	2.45	2.44	2.59	1.87	1.36
Minimum (kb/min)	0.62	0.88	1.12	0.34	0.25
Maximum (kb/min)	4.97	4.65	6.03	6.10	5.42
25% Percentile (kb/min)	2.03	2.01	2.18	1.53	0.80
75% Percentile (kb/min)	2.82	2.82	2.94	2.17	1.65
Number of values measured	238	134	273	583	175
**Asymmetry of replication forks (ratio long/short forks)**					
Median (ratio long/short forks)	1.03	1.03	1.04	1.07	1.10
Mean (ratio long/short forks)	1.07	1.12	1.08	1.19	1.22
Minimum (ratio long/short forks)	1	1	1	1	1
Maximum (ratio long/short forks)	2.03	2.34	1.56	3.77	3.13
25% Percentile (ratio long/short forks)	1.00	1.01	1.01	1.02	1.04
75% Percentile (ratio long/short forks)	1.08	1.10	1.10	1.18	1.24
Number of values measured	104	72	85	112	126
**Number of active replication origins/ haploid genome**					
Size of the haploid genome sequenced*	32.4 Mb	32.8 Mb	32 Mb	26 Mb	2.8 10^3^ Mb
haploid genome/median IOD	180	170	157	175	22.5 10^3^
haploid genome/mean IOD	172	168	142	163	20.6 10^3^

^*^References for genome sequences are Downing *et al.* 2011 for *L. donovani*, Ivens *et al.* 2005 for *L. major*, GeneDB for *L. mexicana*, Berriman *et al.* 2005 for *T. brucei* and NCBI ressources for MEF cells. The figures given for measured values were collected from three or more independent biological experiments.
