# Supplementary material for: Single-molecule analysis of DNA replication reveals novel features in the divergent eukaryotes Leishmania and Trypanosoma brucei versus mammalian cells
Source: Sci Rep. 2016 Mar 15;6:23142. doi: 10.1038/srep23142 (PMC4791591; doi:10.1038/srep23142)
Supplement: Supplementary Information [file srep23142-s1.pdf]

SUPPLEMENTAL MATERIAL

Single-molecule analysis of DNA replication reveals novel features in the divergent eukaryotes *Leishmania* and *Trypanosoma brucei* versus mammalian cells

Slavica Stanojcic, Lauriane Sollelis, Nada Kuk, Lucien Crobu, Yves Balard, Etienne Schwob, Patrick Bastien, Michel Pagès and Yvon Sterkers

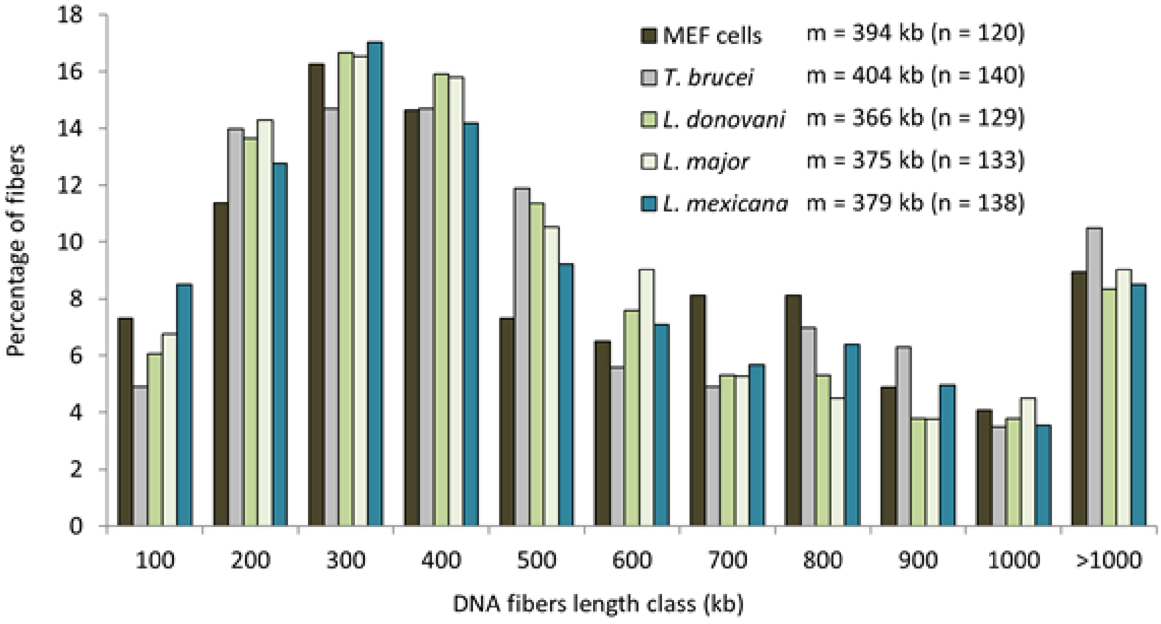

Supplemental Fig 1. Distribution of length classes of DNA fibers in MEF cells, *T. brucei* and three *Leishmania* species.

DNA fibers combed on the coverslips were measured. The median fiber length in kb (m) and the number of measures (n) are indicated for each sample.
